# Nano/Micro-Enabled Modification and Innovation of Conventional Adjuvants for Next-Generation Vaccines

**DOI:** 10.3390/jfb16050185

**Published:** 2025-05-19

**Authors:** Xingchi Liu, Xu Yang, Lu Tao, Xuanchen Li, Guoqiang Chen, Qi Liu

**Affiliations:** 1 School of Engineering Medicine, Beihang University, Beijing 100191, China; xingchiliu@buaa.edu.cn (X.L.); lixuanchen@buaa.edu.cn (X.L.); 2 State Key Laboratory of Biopharmaceutical Preparation and Delivery, Institute of Process Engineering, University of Chinese Academy of Sciences, Chinese Academy of Sciences, Beijing 100190, China; 202261104164@njtech.edu.cn (X.Y.); taolu22@mails.ucas.ac.cn (L.T.)

**Keywords:** nanoparticle, vaccine adjuvants, antigen delivery, microparticle, nano modification

## Abstract

The global spread of infectious diseases has raised public awareness of vaccines, highlighting their essential role in protecting public health. Among the components of modern vaccines, adjuvants have received increasing attention for boosting immune responses and enhancing efficacy. Recent advancements in adjuvant research, particularly nanodelivery systems, have paved the way for developing more effective and safer adjuvants. This review outlines the properties, progress, and mechanisms of FDA-approved conventional adjuvants, focusing on their contributions to and challenges in vaccine success. Despite these advancements, conventional adjuvants still face suboptimal immunomodulatory effects, potential side effects, and limitations in targeting specific immune pathways. Nanodelivery systems have emerged as a transformative approach in adjuvant design, offering unique advantages such as enhancing vaccine stability, enabling controlled antigen release, and inducing specific immune responses. By addressing these limitations, nanocarriers improve the safety and efficacy of conventional adjuvants and drive the development of next-generation adjuvants for complex diseases. This review also explores strategies for incorporating nanodelivery systems into adjuvant development, emphasizing its role in optimizing vaccine formulations. By summarizing current challenges and recent advances, this review aims to provide valuable insights guiding future efforts in designing innovative adjuvants that meet the evolving needs of global immunization programs.

## 1. Introduction

According to the latest report by the World Health Organization (WHO), approximately 8.2 million new tuberculosis cases were confirmed in 2023, marking the highest number recorded since global tuberculosis surveillance began in 1995. Notably, tuberculosis-related deaths in 2023 exceeded those caused by COVID-19. Concurrently, viral hepatitis has emerged as another primary global infectious disease, accounting for approximately 1.3 million deaths annually. Since Edward Jenner’s smallpox vaccine development in the 18th century, vaccines have remained a critical tool in preventing and controlling infectious diseases [1]. Vaccines stimulate the immune system upon administration by activating antigen-presenting cells (APCs), initiating an immune response and conferring protective immunity. Current vaccine platforms include viral vaccines, protein-based vaccines, subunit vaccines, and nucleic acid vaccines [2,3,4]. However, these vaccines face significant challenges in their development and application. For example, vaccines often struggle to provide durable and adequate protection against highly mutable viruses. The emergence of SARS-CoV-2 variants, for example, has diminished the efficacy of COVID-19 vaccines [5,6]. Additionally, vaccines can induce adverse reactions ranging from mild allergic responses to severe side effects. For instance, MMRV vaccines have been linked to seizures, certain mRNA vaccines (e.g., COVID-19 vaccines) to rare cases of myocarditis in young males, and the yellow fever vaccine to neurological inflammatory reactions like encephalitis [7,8,9,10]. Another challenge lies in weak immunomodulatory effects. The hepatitis B vaccine, for instance, typically requires three doses at specific intervals to achieve adequate protection, while post-exposure rabies prevention often necessitates four to five doses. Moreover, populations such as older people and neonates usually exhibit suboptimal immune responses, resulting in insufficient protection [11,12,13]. Vaccine stability also remains a critical issue. Many vaccines require strict storage and transportation conditions, and subunit vaccines are prone to degradation in vivo, which can undermine their effectiveness [14,15].

**Figure 1:**
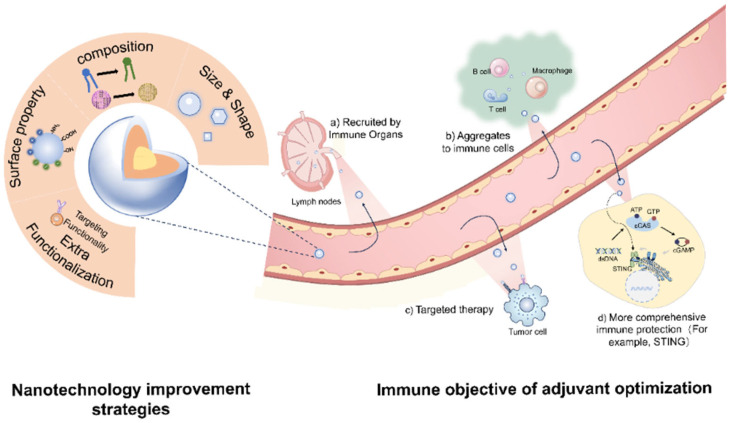
The optimization strategy of adjuvants and the desired immunological objectives.

Modern vaccines typically consist of antigens and adjuvants. Enhancing adjuvants has become a key strategy to address these vaccine limitations. Nanoengineered adjuvants can enhance immunogenicity while minimizing undesirable side effects such as cytotoxicity, primarily through targeted immune cell delivery, controlled antigen release, biocompatible materials, and the optimization of physicochemical properties. By functionalizing the nanoparticle surface with specific ligands, these adjuvants can be selectively directed to antigen-presenting cells (APCs), such as dendritic cells, thereby promoting potent immune activation while reducing off-target effects and limiting cytotoxic responses [16,17]. Moreover, nanoengineered platforms can be designed to release antigens sustainably, emulating natural infection kinetics and thus preventing abrupt immune overactivation and associated tissue damage. Incorporating biocompatible and biodegradable materials—such as lipids, polysaccharides, or synthetic polymers like poly(lactic-co-glycolic acid)(PLGA)—enhances biological safety and facilitates efficient clearance from the body, reducing the likelihood of long-term toxicity or inflammation [16]. In addition, nanoparticles’ size and surface charge are critical in modulating cellular uptake and immune interactions; appropriately tuned characteristics ensure sufficient immune stimulation while mitigating excessive immune activation [18]. This is especially important for vulnerable populations, including the elderly, pregnant individuals, and immunocompromised patients, where precision in immune modulation is vital to avoid adverse outcomes such as autoimmunity, allergic responses, or chronic inflammation. Altogether, these rational design strategies enable the development of nanoengineered adjuvants that provide effective and safe immunostimulation tailored to a broad spectrum of physiological conditions and clinical needs [19]. In addition, different pathogens are recognized by specific pattern recognition receptors (PRRs), such as Toll-like receptors (TLRs), NOD-like receptors (NLRs), and cytokine receptors. By modulating the immune response through adjuvants, vaccines can elicit the most effective form of immunity against diverse pathogens, providing comprehensive protection [20,21]. Incorporating adjuvants enables the immune system to generate robust and tailored immune responses against various pathogens, achieving broader immune protection. Adjuvants are carefully selected during vaccine design to achieve the desired immune outcomes based on the specific antigen components. However, only six vaccine adjuvants have been approved by the FDA for human vaccines (Table 1). This review focuses on three vaccine adjuvants for their wide use, capability, and versatility of modification and antigen co-delivery: aluminum salts, MF59, and immunostimulating complexes (ISCOMs). These elicit distinct types of immune responses and, therefore, hold different application potentials within the field of immunology. Immune responses are generally categorized into the Th1 and Th2 types, with Th1 responses primarily associated with cellular immunity and Th2 responses favoring humoral immunity. However, commercial adjuvants remain inadequate, inducing a well-balanced Th1/Th2 response. Aluminum-based adjuvants, including aluminum oxyhydroxide (AlOOH) and AlPO_4_, tend to skew the immune response toward a Th2 profile, making them suboptimal for inducing Th1-type immunity. These adjuvants primarily activate the NLRP3 inflammasome and are thus better suited for vaccines that enhance antibody production, such as tetanus vaccines. ISCOMs, on the other hand, have been shown to activate both TLR4 and the NLRP3 inflammasome, thereby inducing both Th1 and Th2 immune responses, which makes them favorable for vaccines requiring robust T cell activation, such as antiviral vaccines. MF59 demonstrates a more “neutral” immunological profile by activating TLR2, TLR4, and the NLRP3 inflammasome, making it suitable for vaccines that demand a balance between humoral and cellular immunity. Therefore, vaccine design must consider the desired type of immune response. Th1-type immunity is particularly effective against viral infections, intracellular bacterial pathogens, tumor vaccines, and vaccines requiring long-term protection, whereas Th2-type responses are more suitable for extracellular pathogens and antitoxin vaccines. For most viral, inactivated, and broad-spectrum vaccine strategies, a balanced Th1/Th2 response is often necessary to achieve comprehensive and effective immunological outcomes. Nonetheless, ISCOMs are limited in their ability to encapsulate hydrophilic antigens, often necessitating co-formulation strategies that may reduce antigen uptake efficiency by antigen-presenting cells, while MF59 exhibits weak physical interactions with antigens, compromising its antigen-loading efficiency. Nanodelivery systems offer promising solutions to these challenges by enabling precise control over adjuvant particle size to modulate Th1/Th2 balance, modifying antigen hydrophobicity or hydrophilicity, and adjusting the surface charge of adjuvants to enhance the antigen loading capacity and diversity, thereby achieving more comprehensive and effective immune protection [22,23,24]. Moreover, the antigen–adjuvant loading mode also plays a critical role in shaping the type and magnitude of the immune response. Some adjuvants protect antigens, enabling their slow and sustained release to prolong interaction with immune cells [25,26]. This approach reduces the antigen dosage and lowers vaccine production costs. However, challenges such as adjuvant safety and limited compatibility with specific antigens hinder current adjuvants from fully meeting the demands of modern immunostimulation [27,28]. Consequently, adjuvant development aims to integrate multiple functionalities, elicit more potent immune responses, minimize adverse reactions, and improve vaccine stability. Advances in nanodelivery systems offer promising solutions, providing enhanced safety, stability, and immune efficacy [29,30,31]. Thus, nanoparticle adjuvants are pivotal in optimizing adjuvants and achieving specific immunological objectives (Figure 1).

Nanomaterials, with sizes ranging from 1 to 1000 nm, enhance adjuvant activity by forming potent stimulatory molecular structures. They facilitate efficient antigen transport to immune organs such as lymph nodes (LNs) and the spleen [38,39]. Nanodelivery systems-based engineering methods have demonstrated significant potential in redesigning conventional adjuvants, discovering novel adjuvants, and developing targeted human vaccines. For example, the lipid nanoparticles (LNPs) used in BioNTech’s mRNA COVID-19 vaccine protect mRNA from degradation and enhance cellular uptake, contributing to its global success and marking the first approval of an mRNA vaccine worldwide [40,41,42,43]. Moderna’s mRNA vaccine also employs LNPs to encapsulate nucleoside-modified mRNA encoding the SARS-CoV-2 spike protein for use as a COVID-19 vaccine. Their latest formulation has been authorized for use in individuals aged 6 months to 11 years [44]. The nanoparticle delivery system protects the mRNA from degradation in vivo and facilitates efficient cellular uptake, contributing to the widespread adoption of mRNA vaccines during the COVID-19 pandemic. In recent years, protein-based nanoparticles have also shown promising advancements, particularly in vaccine delivery. Self-assembling protein nanoparticles have been explored for antigen delivery platform for studying the fundamental mechanisms of their interactions with the immune system [45]. Virus-like particles (VLPs) can be engineered to control interactions with their payloads, enabling targeted and efficient antigen release [46]. Furthermore, TLR agonists have emerged as critical components in vaccine development. By stimulating TLR pathways, these ligands can initiate innate and adaptive immune responses, thus addressing the limitations of current adjuvants in achieving comprehensive immune protection. Monophosphoryl lipid A (MPLA), a well-characterized small-molecule TLR4 agonist, has already been approved for use in certain vaccines, such as the human papillomavirus (HPV) vaccine. Leveraging nanodelivery systems to combine such immunostimulatory agents with conventional adjuvants holds great potential for enhancing vaccine efficacy and breadth of protection [47]. Current advances in adjuvant development primarily focus on the following strategies: regulating the timing of antigen exposure to immune cells using nanoadjuvants, enabling stimulus-responsive release through physical or chemical triggers, enhancing the induction of comprehensive immune responses, and achieving targeted delivery to specific immune organs or cell populations. These functional outcomes are intrinsically tied to the physicochemical and structural precision of nanoadjuvants. Nanoengineered adjuvants exhibit several advantageous features: (1) Targeted delivery to immune organs and cells, (2) Activation of broader innate immune responses for long-term protection, (3) Prolonged interaction between immune cells and antigens, improving antigen presentation, and (4) Targeted immunotherapy for cancer cells [48,49,50]. Additionally, certain antigens have benefited from enhancements in nanodelivery systems and have already been successfully applied to some vaccines (Table 2).

To operationalize these strategies, optimizing nanodelivery system-based adjuvants involves precise control over their size and physicochemical properties, including their elemental composition, solubility, hydrophobicity, mechanical properties, surface functional groups, and charge (Figure 1). By modulating the size and shape of adjuvants, it is possible to target specific immune tissues and cells, thereby achieving tailored immunological outcomes [59]. Optimizing the composition of adjuvants is also critical for enhancing immunostimulatory efficacy. Altering certain components can reduce toxicity, improve antigen–adjuvant binding affinity, and enable sustained antigen release, which prolongs immune activation and strengthens memory responses [60]. Furthermore, modifying the surface charge or introducing functional groups can expand the types of antigen interactions, reduce adjuvant aggregation, and improve storage stability, while enabling targeted delivery to specific immune sites [55]. Incorporating additional functional components can stimulate broader immune responses, facilitate targeting of specific immune cells or tissues, and enhance transmembrane and intracellular delivery of antigens [61].

This review examines the limitations of aluminum salt adjuvants, oil-in-water emulsion adjuvants, and key formulations like MF59 and ISCOMs. It further explores the role of nanodelivery systems in modifying and improving these adjuvants, offering potential solutions to address their challenges.

## 2. Aluminum

### 2.1. Overview of Current Research

Aluminum salt adjuvants are the most enduring and extensively utilized immunological adjuvants. A foundational discovery by Glenny et al. in 1926 established aluminum salts’ capacity to potentiate immune responses, heralding the era of aluminum-based adjuvant systems [62]. These adjuvants are chemically categorized into three formulations: aluminum hydroxide (AH), aluminum phosphate (AP), and amorphous aluminum hydroxide phosphate sulfate (AHHPS), with the latter comprising amorphous aluminum hydroxy carbonate and crystalline magnesium hydroxide [63,64].

The immune-potency of aluminum salt adjuvants is governed by four key physicochemical determinants: particle size (1–20 μm range), surface charge, morphological characteristics (fibrous AH vs. lamellar AP structures), and internal crystallinity [65]. Antigen adsorption predominantly occurs through electrostatic interactions dictated by the adjuvant surface charge. AH, with an isoelectric point (pI) of 11.4, exhibits a positive surface charge at physiological pH (7.4), facilitating binding to anionic antigens [66]. Conversely, AP demonstrates a lower pI (4.6–5.6) and carries a net negative charge under physiological conditions, enabling cationic antigen adsorption [67].

While aluminum salt elicits a predominantly Th2-polarized response that enhances humoral immunity and antibody production, its capacity to stimulate the Th1-mediated cellular response remains suboptimal [68]. This limitation poses challenges for vaccines requiring robust cell-mediated protection. Recent advancements in nanostructured materials science have revolutionized adjuvant design, enabling enhanced immunomodulation, improved vaccine efficacy, and reduced side effects. Introducing nanodelivery systems into adjuvant systems presents distinct advantages over conventional aluminum-based formulations, particularly in achieving balanced Th1/Th2 responses [69,70,71].

### 2.2. Nanoalum

While aluminum salts remain widely adopted for their proven safety profile, their inability to elicit cell-mediated immunity limits therapeutic efficacy against malignancies and other conditions requiring Th1 responses [59]. Recent advances in immunology highlight nanoparticle engineering as a promising strategy. Size reduction of aluminum salts from the micron to the sub-micron scale (nanoalum) enhances adjuvant potency, enabling targeted cell-mediated immunity activation [59]. Nanoalum fabrication follows two principal methodologies: top-down and bottom-up approaches.

**Figure 2:**
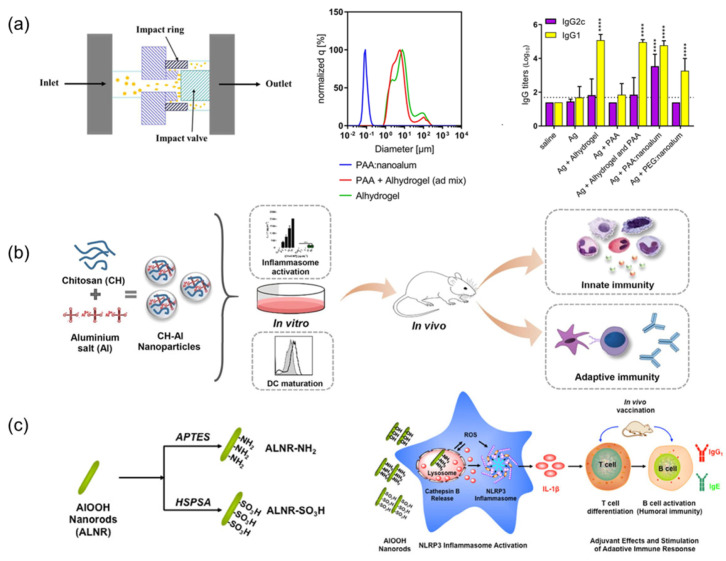
Application of nanodelivery systems in Aluminum Salt Adjuvants: (**a**) Alhydrogel Microflow Nanofabrication: In a study using C57BL/6 mice and the recombinant tuberculosis vaccine antigen ID93, PAA-stabilized nanoalum generated via top-down microfluidization significantly enhanced the Th1-type immune response. The formulation induced high levels of IFN-γ and TNF-producing CD4⁺ T cells, as well as balanced and high-titer IgG1 and IgG2c serum responses. Notably, this effect was achieved without co-administration of TLR4 agonists; Adapted with permission from Ref. [73]. **** *p* < 0.0001 relative to the antigen alone group as determined by one-way ANOVA with Dunnett’s correction for multiple comparisons. (**b**) Chitosan-Doped Aluminum Salt: Using ovalbumin (OVA) as the model antigen in C57BL/6 mice, chitosan-coated aluminum nanorods (CH-NPs) elicited stronger antigen-specific IgG2c responses and a more balanced Th1/Th2 cytokine profile compared to conventional formulations. This enhanced immunogenicity was attributed to improved antigen uptake and inflammasome activation driven by the cationic surface of chitosan-alum hybrids; Reprinted with permission from Ref. [76]. Copyright 2018 Elsevier. (**c**) Surface functionalization of ALNR: In the C57BL/6 mouse model with OVA as the immunogen, cationically modified aluminum oxyhydroxide nanorods (ALNR-NH_2_) promoted higher antigen-specific humoral responses than pristine or SO_3_H-modified ALNRs or conventional alum. The adjuvant activity was linked to elevated ROS generation, lysosomal damage, and activation of the NLRP3 inflammasome; Reprinted with permission from Ref. [55]. Copyright 2017 ACS Publications.

The top-down approach employs high-shear techniques (probe ultrasound/ micro-fluidization) to fragment micro-sized aluminum adjuvants into nanoscale particles [72,73]. Surface stabilization is critical to prevent aggregation—polyacrylic acid (PAA) [73], polyvinylpyrrolidone [72,74], and amino acids [75] demonstrate effective stabilization. Compared to the conventional Alhydrogel, PAA:nanoalum elicited a significantly enhanced TH1 response, with a ~6-fold increase in IgG2c titers (*p* < 0.001) (Figure 2a), a robust CD4⁺ T-cell cytokine profile (IFN-γ⁺/TNF⁺), and markedly elevated serum HAI titers against H1N1 influenza (*p* < 0.01). Notably, 100% of mice vaccinated with nanoalum survived a lethal influenza challenge versus 60% with Alhydrogel and 40% with the antigen alone (*p* < 0.01, Log-rank test) [73]. However, despite these immunological advantages, top-down nanoalum formulations face significant challenges in scalability and reproducibility. Re-agglomeration during storage—particularly when co-formulated with negatively charged adjuvants like CpG 1018—remains a major concern, compromising batch-to-batch consistency [73]. To address this, strategies like increasing the CpG-to-aluminum ratio or incorporating small-molecule polyanions (e.g., phytic acid) have demonstrated efficacy in maintaining nanoscale dispersion and inhibiting time-dependent particle growth. Although top-down manufacturing approaches are compatible with industrial-scale equipment such as micro-fluidizers, precise control over shear forces, processing temperature, and excipient concentrations is crucial to ensure product uniformity. To further improve scalability and formulation robustness, the integration of in-line process analytical technologies—such as real-time particle size analysis and zeta potential monitoring—can provide continuous quality assurance during production. Additionally, freeze-drying with protective excipients offers a viable method to prevent storage-induced aggregation, thereby enhancing long-term stability.

Bottom-up synthesis involves controlled precipitation of aluminum ions under alkaline conditions, facilitated by stabilizers like graphene oxide and PEG-poly (AGE-Suc) [77,78]. Graphene oxide’s surface functional groups (carboxyl, carbonyl, hydroxyl) mediate Al-OH complexation, while anionic PEG-poly (AGE-Suc) electrostatically encapsulates aluminum hydroxide particles [77,78]. Li et al. developed Mg/Al-LDH nanoparticles (102.9 nm) via hydrothermal synthesis, demonstrating enhanced DC-mediated antigen uptake and a humoral response comparable to a commercial AH adjuvant, with reduced local inflammation. Compared with a conventional aluminum adjuvant (Alhydrogel), the Mg/Al-LDH nano-adjuvant demonstrated superior performance across multiple key indicators: Animal experiments showed that the Mg/Al-LDH-PTd (10:1) group elicited significantly higher anti-pertussis toxin (PTd) IgG antibody levels on both Day 21 and Day 35 compared to the unadjuvanted PTd group (* *p* < 0.05, ** *p* < 0.01, n = 6), with titers comparable to those induced by Alhydrogel, but with a more rapid onset of humoral response. Regarding immune polarization, both Mg/Al-LDH and Alhydrogel significantly increased IgG1 levels (Th2-type, ** *p* < 0.01), while no significant enhancement in IgG2a (Th1-type) levels was observed. This indicates that both adjuvants promoted a Th2-skewed humoral immune response [79]. This lamellar structure enables sustained antigen release and improved lymphatic trafficking, while nanoscale dimensions promote TLR-mediated innate activation and cross-presentation. Nonetheless, bottom-up methods—despite offering excellent control over particle size and crystallinity—face significant barriers in industrial translation due to their high sensitivity to reaction parameters such as pH, temperature, and reagent addition rates. These factors complicate scale-up and often result in poor batch-to-batch reproducibility.

To overcome these challenges, bottom-up synthesis strategies could be improved by integrating automated platforms capable of maintaining precise environmental control and real-time feedback. Additionally, high-throughput screening of precipitation conditions can help identify robust operational windows, enhancing process reliability. Post-synthetic surface modifications, such as grafting with inert polymers, may further provide steric stabilization, reduce inter-batch variability, and improve long-term storage stability.

Crystallographic engineering further modulates immuno-stimulation. Sun et al. engineered AlOOH nanostructures showing crystallinity-dependent NLRP3 inflammasome activation [80]. High-aspect-ratio nanorods exhibit preferential DC uptake and lysosomal disruption, driving IL-1β production via optimized surface hydroxyl density. Low-crystalline variants enhance antigen adsorption through increased chemical reactivity, whereas highly crystalline forms demonstrate reduced immune activation due to structural inertness.

Mechanistically, nanoalum overcomes conventional alum’s Th2 bias through size-dependent cellular processing. Micron-scale particles (1–20 μm) remain injection-site localized, mediating macrophage-dependent humoral responses. In contrast, nanoalum (<1 μm) facilitates APC endocytosis, lysosomal activation, and subsequent Th1 polarization [81,82,83,84]. Practical advantages include simplified sterilization—nanoalum maintains physicochemical stability under autoclaving (121 °C, 30 min), unlike traditional alum that requires upstream sterilization [85].

Although in vitro immunostimulatory profiles are well-documented, compelling in vivo evidence also substantiates the immunological benefits of nanoformulated aluminum adjuvants. For example, Liang et al. designed a novel combination adjuvant by chemically conjugating CpG oligodeoxynucleotides (ODNs) with AlOOH nanorods, exploring the effect of CpG orientation (5′ vs. 3′ exposure) on immune responses. Using both hepatitis B surface antigen (HBsAg) and SARS-CoV-2 RBD as model antigens, they demonstrated in vivo that Al-CpG-5′ significantly enhanced both humoral and cellular immunity, inducing stronger Th1 and Th2 cytokine responses, greater lymph node transport, and higher titers of IgG subclasses compared to traditional Al + CpG mixtures [57]. In a related study, Bi et al. constructed AlOOH nanorods with varying aspect ratios to modulate adjuvant properties. They found that high-aspect-ratio nanorods (NR4) promoted greater dispersion, more substantial antigen uptake by macrophages, and elevated IL-6 secretion, ultimately enhancing Th2 dominant antibody responses to HBsAg in mice [86]. Additionally, Sun et al. systematically investigated the role of AlOOH nanorod crystallinity and shape in NLRP3 inflammasome activation. Their studies using an OVA vaccine model revealed that nanorods with lower crystallinity and a higher hydroxyl content triggered greater ROS generation and IL-1β production, leading to amplified adaptive immunity. Collectively, these studies underscore the importance of nanoengineering aluminum adjuvants in enhancing both innate and adaptive immune responses by controlling structural parameters and CpG orientation [80].

### 2.3. Composite Adjuvant Nano-Aluminum Salts

Metal-organic frameworks (MOFs), characterized by their porous structures that are formed through metal–ligand coordination, are increasingly explored in biomedical contexts, including vaccine adjuvant design [87,88]. Zhong et al. pioneered a biomimetic mineralization approach to synthesize Zeolitic Imidazolate Framework-8 (ZIF-8)-encapsulated aluminum nanoparticles (ZANPs), achieving efficient antigen (OVA) loading via imidazole-mediated hydrophobic interactions and hydrogen bonding [89]. This composite system capitalizes on ZIF-8’s pH-responsive degradation coupled with AlO(OH)-induced lysosomal destabilization, synergistically enhancing MHC I antigen presentation and CTL activation. In EG7-OVA tumor-bearing mice, CpG/ZANPs exhibited remarkable tumor suppression through concurrent NLRP3 inflammasome activation and cGAS-STING-dependent type I interferon (IFN) production, effectively overcoming conventional alum’s Th1 response limitations [89]. Chitosan–aluminum hybrids present another promising strategy. Lebre et al. developed chitosan–aluminum nanoparticles (CH–Al NPs) that stimulate NLRP3-mediated IL-1β secretion while maintaining alum-level IgG production with improved safety profiles (Figure 2b) [76]. Liu’s subsequent work with N-2-hydroxypropyltrimethylammonium chitosan nanoparticles (N-2_HACC NPs) (300 nm, ζ = +32.23 mV) demonstrated enhanced avian vaccine performance, evidenced by 2.3-fold higher IFN-γ levels compared to commercial formulations and superior APC activation [90].

The clinical success of the AS04 adjuvant system (alum + MPLA) in HPV/hepatitis B vaccines underscores the potential of combining aluminum salts with immunostimulants [91,92]. This approach leverages TLR4 activation to potentiate Th1 responses while preserving alum’s humoral immunity advantages. Similarly, aluminum-imiquimod combinations enhance DC activation through multimodal pattern recognition receptor engagement.

Emerging nanocarrier applications address traditional aluminum adjuvants’ cellular immunity constraints through precise nano-structuring (<200 nm), surface charge modulation, and MOF integration. These innovations enable enhanced lymphatic targeting, multivalent immune pathway activation, and thermostable formulations compatible with standard sterilization protocols. The convergence of materials science and immunology thus opens new frontiers in rational vaccine design.

### 2.4. Surface Modification

Nanocarrier-driven surface engineering enhances aluminum salt adjuvants’ immunological performance through targeted modifications. Emerging strategies demonstrate the potential for optimizing conventional adjuvant systems via rational surface functionalization.

Sun et al. engineered AlOOH nanorods (ALNRs) with amine (-NH_2_) or sulfonic acid (-SO_3_H) groups (Figure 2c). Amine-functionalized ALNRs (ALNR-NH_2_) exhibited superior cellular internalization, lysosomal disruption, and NLRP3 inflammasome activation, correlating with enhanced IL-1β secretion and antibody titers compared to sulfonated or unmodified counterparts [55]. Polyethylene glycol (PEG) derivatives further exemplify surface modification benefits. Using PEG-derived PPAS to prepare aluminum hydroxide-based nanovaccines (APNs) and conjugating them with the antigen and zoledronic acid (ZOL), Sun’s team developed APN-OVA-ZOL nanoparticles that enhanced lymph node targeting and MHC-I antigen presentation through optimized DC uptake. Moreover, the prepared APNs exhibited excellent stability, as evidenced by negligible changes in their hydrodynamic diameter during one week of storage at 4 °C [93,94]. Antigen adsorption can be augmented via phosphate group introduction (pSer/C-PO3), leveraging ligand exchange with aluminum hydroxide surfaces [95]. Surface hydroxyl density critically influences antigen adsorption kinetics and structural preservation. Higher hydroxyl content correlates with stronger antigen binding and prolonged release profiles, though potential antigen conformational changes warrant structural stability assessments [96]. Although the functionalization of aluminum-based adjuvants has led to certain improvements in immunogenicity, studies on the stability of these modified adjuvants—particularly under physiological conditions—remain lacking. Such stability assessments are crucial for evaluating the efficacy and safety of functionalized adjuvants.

Chen et al. developed hyaluronic acid-coated aluminum hydroxide nanoparticles (B/O@AN, 10-100 nm) encapsulating Beclin-1 peptide and ovalbumin. This design promoted DC maturation through autophagy-mediated antigen cross-presentation, amplifying Th1/CTL responses and inhibiting tumor growth [97].

Metal ion integration expands adjuvant functionality [98,99,100]. Liu’s manganese-modified aluminum (Mn-Al) adjuvant activated cGAS-STING signaling while enhancing antigen adsorption, demonstrating dual humoral/cellular immunity enhancement [101]. CpG–aluminum conjugates show orientation-dependent efficacy: 5′-exposed CpG (Al-CpG-5′) outperformed 3′-exposed variants in DC activation and Th1/Th2 cytokine production, with elevated CTL markers and IgG2a titers [57].

This surface modification enables three key advancements: (1) controlled antigen-adjuvant interactions through functional group engineering, (2) synergistic effects with immunostimulatory coatings (e.g., autophagy inducers), (3) emergent immunomodulatory properties via metal ion coordination. These approaches overcome traditional alum’s Th2 bias while maintaining safety profile, establishing nanoparticle adjuvants as a transformative tool in next-generation adjuvant design.

Aluminum-based adjuvants have been widely used due to their favorable safety profile; however, they primarily elicit Th2-type immune responses. Although the integration of nanodelivery systems has led to some improvements, clinical progress remains limited, making them less suitable for vaccines that require strong cellular immunity. In contrast, ISCOMs have demonstrated superior efficacy in inducing Th1-type responses. The following section will provide an overview of ISCOMs.

## 3. ISCOMs

### 3.1. Overview of Current Research

ISCOMs are self-assembled nanoparticles comprising saponins (primarily Quil A derivatives), cholesterol, phospholipids, and antigens. Saponins—naturally occurring glycosides with inflammatory and antitumor effects—serve as the key immunostimulatory component by activating mammalian immune pathways [102,103]. Synthesized ISCOMs exhibit spherical cage-like structures (40–50 diameters) with porous surfaces, optimizing cellular uptake while stabilizing Quil A and mitigating its cytotoxicity [104,105]. Mechanistically, ISCOMs promote antigen trafficking to drain LNs via mast cell-dependent lymphatic modulation and enhance DCs-mediated cross-presentation for robust CD8^+^ T cell priming [106,107].

**Figure 3:**
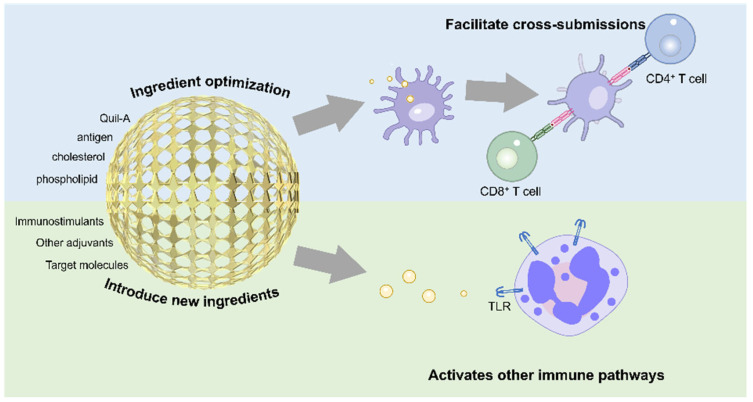
Key improvement strategies for ISCOMs are presented, along with a simple diagram illustrating their enhanced cross-presentation capability.

First described by Morein et al. in 1984 [108], ISCOMs have evolved into widely used veterinary vaccines in Europe and North America, demonstrating efficacy in swine, equine, and poultry immunization [109,110]. Cuevas-Romero et al. immunized pregnant sows with ISCOMs-adjuvanted rHN-PorPV, achieving 87.5% and 75% survival rates in piglets against porcine rubulavirus versus 100% mortality in controls [111]. ISCOMs stimulate localized inflammation through osteopontin-mediated neutrophil/macrophage recruitment and upregulation of chemokine networks (CCL2, CCL19, CXCL2, CXCL16, CCR5), enhancing APC aggregation [112,113]. Despite these advances, clinical translation faces challenges: Quil A exhibits dose-dependent cytotoxicity (hepatic degeneration in murine models), while complex fabrication protocols hinder scalable production [103,114,115,116]. Current optimization strategies focus on compositional refinement and nanoscale engineering to improve safety and manufacturability (Figure 3).

### 3.2. Nanostructure Modification Improvement

Component optimization enables ISCOM enhancement without altering their core structure [103]. QS-21—a purified Quil A fraction with improved stability—shows promise in malaria and cancer vaccines [60,117]. Alternatively, different purified saponin components can be incorporated into the same ISCOM to achieve the saponin content required to elicit an immune response. As a structurally complex saponin, the mechanisms underlying the synergistic actions of the various components of Quil A remain poorly understood. Due to its potential hemolytic activity, it may cause a certain degree of hepatocellular damage [118]. Studies have shown that the cytotoxicity of Quil A, ranging from 20% to 70%, does not necessarily increase with higher concentrations [119]. This phenomenon is attributed to the increased hydrophilicity and enhanced negative surface charge of the particles at higher concentrations, which reduces their cellular uptake efficiency. Moreover, ISCOMs containing 20% Quil A have been observed to elicit stronger cell activation, indicating that precise dosage control is particularly critical for saponin-based adjuvants to achieve optimal immune stimulation [119]. Besides dose-dependent toxicity assessments and dosage control, nano-based strategies may be employed to mitigate the cytotoxicity of Quil A, such as structural modification to reduce its direct interaction with cell membranes or encapsulation within nanoparticles to enable sustained release, thereby minimizing cellular toxicity while preserving immunostimulatory efficacy. Moreover, the individual components of Quil A exhibit varying effects on biological systems; for instance, QS-18, in organisms different from those of Quil-A, is more toxic than Quil A. Research focuses on exploring ISCOMs formed by different saponin components to ensure they exhibit good safety and strong immunomodulatory effects. Matrix-M, blending low-activity Fraction-A and high-potency Fraction-C saponins, achieved dose-sparing efficacy in NVX-CoV2373’s Phase III COVID-19 trials [120,121,122,123,124]. Saponin-based adjuvants have been challenging to apply in human vaccines due to their complex composition and the uncertainty of their effects. In future research, elucidating the impact of individual components and their interactions will be a key approach to advancing the development of saponin-based adjuvants.

Phospholipid engineering further improves stability. Replacing lecithin with distearoylphosphatidylcholine (DSPC) or other long-chain saturated phospholipids enhances structural integrity [125], while fatty acid unsaturation inversely correlates with thermal transition temperatures [126]. Charge modulation via 1,2-dioleoyl-3-trimethylammonium-propane (DOTAP) substitution or cationic cholesterol derivatives (e.g., 3β-N-(N′, N′-dimethyl aminoethane)-carbamoyl cholesterol) enhances APC membrane interaction and internalization [127,128].

Optimizing and adjusting the components of ISCOMs enhances their stability and safety while boosting their immunomodulatory effect. Exploring the roles of individual Quil A components and their synergistic effects could improve immunostimulants while reducing toxicity. Similarly, altering the properties of ISCOM phospholipids and cholesterol can modify their charge characteristics and structural stability. These adjustments leverage nano-based strategies to refine the structural and functional properties of ISCOMs, tailoring them to achieve the desired adjuvant properties. Future research could explore incorporating lipids with additional functionalities to enhance their immune-stimulating effects, thereby gaining more comprehensive immune protection. These modifications and improvements hold significant promise for advancing ISCOM optimization.

### 3.3. Nano-Functionalized Modification

Functional modifications of ISCOMs aim to broaden antigen compatibility and enhance immunological precision. While native ISCOMs preferentially incorporate hydrophobic antigens [129,130,131], the engineered ISCOMATRIX platform enables hydrophilic antigen loading through two primary strategies: (1) electrostatic coupling via antigen fusion with cationic amino acids [132,133] and (2) metal chelation anchoring using phospholipid–metal ion conjugates [134]. Using Balb/c and C57Bl/6 mice as well as New Zealand White rabbits as models, Buglione-Corbett et al. demonstrated ISCOMATRIX’s capacity to enhance DC-mediated antigen presentation in an HIV vaccine model, eliciting robust CD4^+^/CD8^+^ T-cell responses alongside Th1/Th2 cytokines as well as chemokines (G-CSF, KC, and MIP-1α) [135].

Targeting specificity has been improved through the covalent lipid conjugation of glycoproteins/peptide ligands [136,137,138]. By leveraging the selective reactivity of vinyl sulfone with protein amino/thiol groups under physiological conditions, researchers grafted protein A onto ISCOMs via hydrophobic anchors. This modular system enables targeted delivery through functionalized nano-capsules [61]. Mucosal applications demonstrate versatility: intranasal administration of cholera toxin B (CTB)-conjugated ISCOMs synergistically enhances distal reproductive tract IgA responses, underscoring mucosal immunization potential.

Adjuvant synergy expands the immunological scope. McCluskie et al. augmented ISCOMATRIX with CpG ODN, achieving the Th1-polarized IFN-γ responses critical for combating chronic infections and malignancies [139]. This combinatorial approach exemplifies how nano-structural engineering integrates complementary immune activators (e.g., TLR4 agonist MPLA) to achieve multipronged immune responses [139].

Recent advancements employ Box–Behnken experimental designs to optimize neuropeptide Y (NPY)-loaded ISCOMs. These formulations demonstrate enhanced encapsulation efficiency alongside tumor-targeted immunomodulation, with reduced TNF-α levels and increased IL-10 levels, revealing dual functionality as anticancer agents and immune regulators. Such innovation highlights ISCOMs’ expanding therapeutic potential beyond conventional vaccine applications.

ISCOMs are currently used predominantly in veterinary vaccines, and concerns regarding their potential toxicity remain. Future integration with nanodelivery systems is hoped to enable the broader application of ISCOMs in human vaccines. In contrast, oil-in-water emulsion adjuvants have found wider use in human immunization. The following section focuses on MF59 as a representative example.

## 4. MF59

### 4.1. Overview of Current Research

MF59 is an oil-in-water emulsion adjuvant with an average particle diameter of ~160 nm. Its oil phase consists of squalene, a naturally occurring triterpene hydrocarbon involved in cholesterol biosynthesis and vitamin D production. Squalene enhances antigen delivery by promoting antigen attachment to APCs like DCs and macrophages and inducing localized inflammation to amplify the immune response [140,141,142]. The formulation further includes two non-ionic surfactants, Tween 80 and Span 85.

MF59 originated during Chiron Vaccines’ development of a hepatitis B recombinant vaccine, where alum-based formulations showed insufficient immunomodulatory effects. This led to the creation of MF59 as a delivery system. Subsequent preclinical studies revealed its intrinsic adjuvant properties [143]. Following refinement, MF59 received European approval in 1997 for use in enhanced influenza vaccines targeting individuals aged 65 and above [144,145,146].

MF59 activates local immune responses by stimulating chemokine secretion (CCL2, CCL4, CXCL8) to recruit immune cells while enhancing monocyte endocytosis and upregulating MHC class II/ CD86 expression [147,148]. In mature DCs, it elevates CD83 and CCR7 expression, driving robust immune activation and cellular recruitment [149].

Approved in more than 30 countries for influenza vaccines, MF59 remains critical for novel vaccine development, including SARS-CoV-2 candidates. A Phase I trial by Chappell et al. demonstrated that an MF59-adjuvanted spike protein clamp vaccine induced potent neutralizing antibodies and a Th1-polarized CD4^+^ T-cell response. However, two doses were required for optimal immune responses [150]. However, MF59 typically exhibits weak physical interactions with antigens. As an oil-in-water emulsion, MF59 consists of squalene oil droplets stably dispersed in an aqueous phase. Due to the hydrophobic nature of its droplet surface and the lack of strong affinity interactions—such as electrostatic adsorption or coordination bonding—with antigens, its capacity to serve as an effective antigen delivery system is limited. Furthermore, the presence of surfactants on the droplet surface hinders direct binding between the oil droplets and antigens. Additionally, the primary component of MF59, squalene, possesses a relatively low antioxidant capacity, raising concerns regarding the formulation’s long-term stability. These factors collectively highlight some of the inherent limitations of MF59 as an adjuvant system, which highly necessitates nano-based engineering strategies [150].

### 4.2. Nano-Structural Adjustment

**Figure 4:**
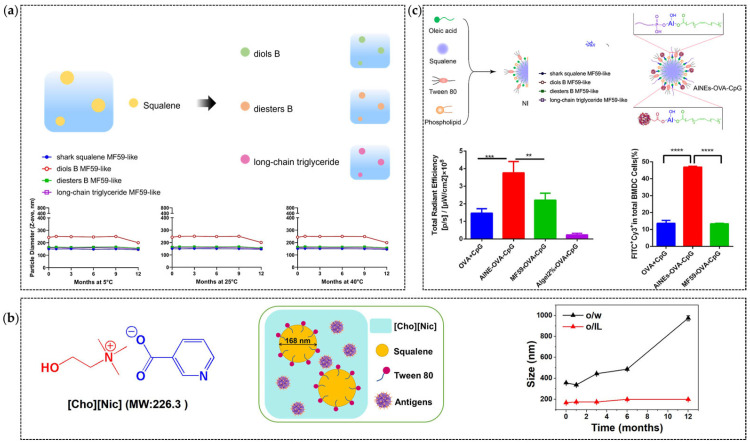
Applications of Nanodelivery Systems in Emulsions: (**a**) Semi-synthetic terpenoids with different adjuvant properties can be used as an alternative oil phase of squalene [154]; Adapted with permission from Ref. [154]. (**b**) Enhancing the Uniformity and Stability of MF59 by Using [Cho][Nic] as a substitute for the aqueous phase [152]. The in vivo protection results are tested on the BALB/c mice mouse model, and the mice were immunized with a split influenza virus as the antigen and MF59 by injection on Days 0, 14; Adapted with permission from Ref. [152]. Copyright 2022 Elsevier (**c**) Incorporating aluminum hydroxide into MF59 to enhance antigen binding capacity in the C57BL/6 mouse model by using OVA as model antigen by injection on Days 0, 7 and 14 [155]. Adapted with permission from Ref. [155]. Copyright 2022 Elsevier. ** indicate “*p* < 0.01”, *** indicate “*p* < 0.001”, **** indicate “*p* ≤ 0.0001”.

Nano-structural optimization has enhanced MF59’s stability and immunostimulatory performance. Squalene’s oxidative instability necessitates oil-phase modification [151]. Researchers have tested the stability of several potential squalene substitutes after synthesizing MF59 (Figure 4a). Yuan et al. developed squalene–aluminum stabilized emulsions (ASEs) prepared with soybean, peanut, and olive oils with varying oleic/linoleic acid (O/L) ratios. Soybean-based ASEs (O/L ratio = 1:2.75) demonstrated superior long-term stability and charge retention [23]. These emulsions were prepared in a single step using ultrasonication with aluminum hydroxide. Lin et al. created an oil-in-ionic liquid (o/IL) emulsion using choline–nicotinic acid ionic liquid ([Cho][Nic]), squalene, and Tween 80 (Figure 4b). The formulation achieved a 168.6 nm diameter, a PdI of 0.068 and 12-month stability at 25 °C via reduced interfacial tension. Following intranasal mucosal immunization in mice, o/IL significantly enhanced the prolonged retention of the antigen in the nasal cavity and promoted its paracellular transport across the mucosal epithelium. As a result, the secretory IgA titer in mice was 25-times higher than that induced by the bare antigen and 5.8-times higher than that achieved with the commercial MF59-adjuvanted antigen [152]. Wang et al. replaced squalene with vitamin E, yielding lipid droplets (LDs) stable under 120 °C sterilization, underscoring oil-phase optimization’s impact on thermal resilience [153].

Surfactant-free approaches offer alternative stabilization strategies. Xia et al. developed PLGA-stabilized raspberry-like PPAS emulsions, where interparticle gaps enabled three-site antigen stabilization via hydrogen bonding. Moreover, the absence of surfactants allows the formulation to adopt a raspberry-like morphology, providing a large specific surface area for antigen adsorption and cell interaction. Compared to the conventional structure of MF59 adjuvants, this design strategy offers improved antigen-binding capacity and increases the number of potential sites for cellular engagement, thereby enhancing overall immunogenicity. Additionally, the absence of surfactants in this method eliminates the possible risks commonly introduced by such agents in conventional emulsions. Comparative experimental data indicate that relative to PPAS, traditional emulsions demonstrate elevated cytotoxicity at higher concentrations [156]. PPAS enhanced CD86 expression at injection sites and amplified Th1/Th2 responses, achieving 304% and 278% increases in IFN-γ-secreting CD8^+^ T cells and SINFEKL-MHC I^+^ CD8^+^ T cells, respectively, versus antigen alone [156]. This strategy offers an innovative approach to improving the binding capacity between emulsion adjuvants and antigens. By eliminating surfactants, the emulsion’s specific surface area increases, allowing for the adsorption of more antigens, while the added stabilizers help retain and stabilize the captured antigens. These findings demonstrate the feasibility of this strategy for enhancing emulsion adjuvants. Additionally, exploring other stabilizers could further improve the antigen adsorption capacity.

Particle size critically influences immune targeting [157,158]. In terms of delivery, nanoparticles offer certain advantages over microparticles, such as enhancing cellular uptake, improving the bioavailability of poorly soluble drugs, and optimizing pharmacokinetic properties [159]. For example, 20 nm particles localize to splenic B-cell-rich zones, while 500–1000 nm particles accumulate in DC-rich regions [160]. Nanoscale squalene emulsions (MF59–0, 214 ± 54 nm) include higher antibody titers than microemulsions (K59–0, 1080 ± 360 nm), emphasizing size-dependent efficacy [161].

### 4.3. Optimization of Antigen Delivery Strategies

While MF59 excels in influenza and COVID-19 vaccines, its weak antigen binding limits delivery efficiency [162]. In contrast, aluminum salt adjuvants can effectively bind antigens through electrostatic attraction and “ligand exchange” [163]. Inspired by this, Chen et al. addressed MF59’s limitations by coating an MF59 nanoemulsion with aluminum hydroxide (AlNEs) (Figure 4c). This system was further functionalized by adsorbing antigens and the immune enhancer CpG onto its surface, creating AlNEs-OVA-CpG. This system enhanced APC maturation (CD40/CD80/CD86 upregulation) and lymph node accumulation while maintaining safety [155].

In addition to increasing the antigen load in MF59, researchers have found that chitosan, as a coating material for drug delivery carriers, can enhance the APC uptake of vaccines [164]. Chitosan-modified squalene-based cationic nanostructured lipid carriers (csNLCs) further improve APC uptake via charge-mediated endocytosis, boosting antigen-specific IgG and IL-4 responses [164]. Surface charge modulation represents a promising strategy for tailoring immune outcomes, with cationic formulations showing enhanced APC uptake and lymphocyte activation.

## 5. Conclusions and Outlook

In this article, we reviewed the components, structural characteristics, and existing limitations of three types of adjuvant–aluminum salts, ISCOMs, and MF59—alongside nanodelivery system applications in adjuvant design. While traditional adjuvants exhibit well-documented safety profiles and remain integral to current vaccines, modifications of these established adjuvants remain essential. Table 3 presents clinical-stage applications of optimized traditional adjuvants in emerging vaccine candidates. Nanodelivery systems, as a rapidly evolving interdisciplinary field, demonstrate dual significance: enhancing conventional adjuvants through structural refinement and enabling synergistic integration with novel nanomaterials—a critical advancement for next-generation adjuvant development. Future innovations in traditional adjuvant modification will increase the leverage of nanodelivery systems at the microstructural level. Precise control of adjuvants’ size and shape/morphology can yield optimized formulations. Simultaneously, the strategic introduction of functional groups or bioactive compounds may enable novel immunostimulatory mechanisms or multifunctional applications, including immune diagnostics and labeling.

Nanomaterial advances are driving the exploration of inert and inorganic nanoparticles as potential replacements for conventional adjuvants and antigen-delivery systems [171,172,173]. Integrating these nanomaterials with traditional adjuvants could yield hybrid systems with enhanced safety and stability. Nanomaterials may redefine adjuvant–antigen interactions, enabling improvements such as precise antigen delivery in MF59-based systems, amplifying adjuvant immunostimulatory capacity for stronger protection, or nanoparticle-mediated targeting to enhance vaccine specificity and delivery efficiency.

**Figure 5:**
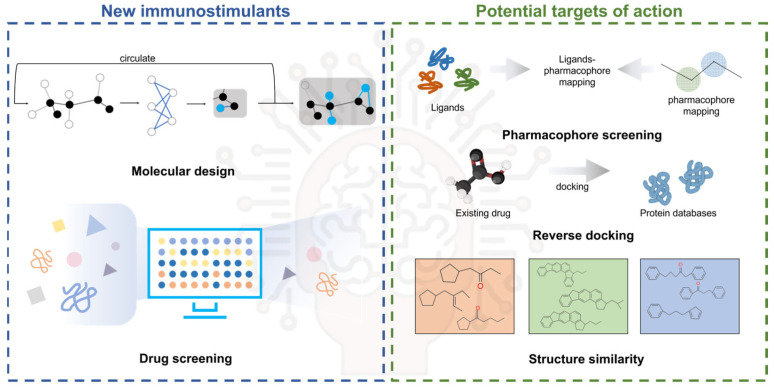
AI assists in the discovery of new immune targets and the screening and discovery of immunostimulants.

Concurrently, artificial intelligence (AI) is emerging as a transformative tool revolutionizing biomedical research, particularly in advancing immunotherapy and adjuvant development. In the context of novel nanomaterials and modified adjuvants, comprehensive nanotoxicological profiling necessitates the multi-scale modeling of adjuvant–cell–organ interactions through molecular dynamics simulations and predictive cellular models to evaluate cytotoxic behavior. High-throughput screening (HTS) has proven indispensable for the rapid biocompatibility assessment and toxicity profiling of emerging nanomaterials, enabling the efficient identification of adjuvant-compatible candidates. Concurrently, AI-driven methodologies demonstrate transformative potential in three key domains: (1) immune-target discovery through pattern recognition in complex datasets, (2) structure–function prediction of immunostimulant via deep learning architectures, and (3) pharmacokinetic modeling to optimize adjuvant delivery dynamics (Figure 5). The systematic application of AI has enabled the mechanistic elucidation and structural optimization of conventional adjuvant systems. By combining molecular dynamics simulations with expanding compound libraries, deep learning algorithms help identify novel agonist motifs that enhance immunostimulatory efficacy. This computational framework further guides the structural refinement of existing adjuvants through predictive toxicity reduction strategies, while maintaining or augmenting therapeutic potency. Such AI-enabled modifications significantly improve biocompatibility and functional performance precision, addressing critical barriers to clinical translation in vaccine development. The strategic integration of AI with conventional adjuvant research represents a part-paradigm-shifting approach to next-generation adjuvant design. This synergy accelerates structure–activity relationship analysis and establishes predictive frameworks for rational adjuvant engineering, ultimately advancing the development of safer and more effective human vaccine formulations.

In addition, the scalability and feasibility of nanodelivery systems remain significant challenges. Currently, nanoparticle formulations are primarily developed under tightly controlled laboratory conditions, but their consistency and efficiency tend to decline when scaled up for mass production. Moreover, the equipment required for large-scale manufacturing is often costly, making widespread production difficult. Storage and stability issues also persist—for instance, mRNA is prone to structural degradation during storage and transport, with some vaccines requiring ultra-cold conditions (e.g., −70 °C), which severely limits their broad deployment [174]. Despite the significant advantages of nanomaterial-based adjuvants in enhancing immunogenicity, their long-term or repeated exposure may pose considerable safety concerns [175,176]. Key issues include chronic inflammation resulting from sustained NLRP3 inflammasome activation, immune dysregulation due to Th1/Th2 imbalance and Treg dysfunction, nanoparticle accumulation in immune organs (e.g., liver, spleen, lymph nodes) that alters local immune environments, increased risk of hypersensitivity from excessive IgE production, and oxidative stress caused by the prolonged generation of reactive oxygen species (ROS), which can impair immune cell function [81]. To address these risks, several mitigation strategies have been proposed, including the design of biodegradable and rapidly excretable nanomaterials, the use of stimuli-responsive or controlled-release systems to avoid chronic stimulation, surface engineering to reduce unintended immune activation, and the integration of long-term in vivo studies and immunotoxicity profiling early in adjuvant development. These approaches aim to strike a controlled balance between enhanced immune efficacy and long-term safety.

In summary, adjuvants remain indispensable vaccine components, with traditional formulations maintaining higher public acceptance. Nano-engineering approaches to adjuvant optimization provide viable strategies to address efficacy and safety concerns. To meet demands for durable protection and novel target engagement, systematic investigation of adjuvants’ structure–activity relationships—elucidating their mechanisms of action and toxicity profiles—must be prioritized in the AI era. Moreover, nanotechnology has been leveraged to improve vaccine stability, addressing challenges in formulation, storage, and transportation. Next-generation vaccine adjuvants could achieve global implementation through such integration, while sustaining their vital role in safeguarding public health.

## Figures and Tables

**Table 1:** FDA-Approved Vaccine Adjuvants for Human Use.

Name	Composition	Activation Mechanisms and Interactions with the Immune System	Vaccine Application	Limitations	Reference
Aluminum Adjuvant	Aluminum phosphate, aluminum hydroxide, aluminum potassium sulfate, amorphous aluminum phosphate, etc.	Th2 humoral immune response induction by activating NLRP3 inflammasome	Widely used in vaccines for diphtheria, tetanus, pertussis, Haemophilus influenza type B, pneumococcal, hepatitis A, hepatitis B, etc.	Th2-biased, low antigen-binding capacity, prone to aggregation	[32]
AS04	Combination of 3-O-deacyl-4′-monophosphoryl lipid A (MPL) and aluminum salt	Th1/Th2 immune response induction by activating NLRP3 inflammasome	Cervarix for preventing HPV and Fendrix for preventing hepatitis B	Weak Th1 activation, poor receptor synergy	[33]
MF59	Oil-in-water emulsion made of squalene, polysorbate 80 (Tween 80), and sorbitan trioleate (Span 85)	Th1 and Th2 induction via ATP release from muscle fibers, initiating CD4⁺ T-cell priming and NLRP3 inflammasome activation	Influenza vaccines, particularly for seasonal and pandemic flu in the elderly	Low antigen affinity, poor stability	[34]
AS03	Oil-in-water emulsion containing DL-α-tocopherol, squalene, and polysorbate 80 (Tween 80)	Th1 and Th2 induction via NF-κB pathway and upregulation of inflammatory cytokines such as IL-6 and TNF-α	H1N1 influenza vaccines and RTS, S malaria vaccine	Harsh prep, unstable, local toxicity	[35]
AS01b	Liposome containing 3-O-deacyl-4′-monophosphoryl lipid A (MPL) and saponin QS-21	Th1 induction via TLR4 stimulation on dendritic cells (DCs)	Shingrix for shingles and Mosquirix for malaria	Local/systemic toxicity, QS-21-linked risk, challenging for large-scale manufacturing	[36]
CpG1018	CpG oligodeoxynucleotide (TLR9 agonist)	Th1 induction via TLR9-triggered DC maturation	Heplisav-B for hepatitis B	No self-delivery, carrier-dependent	[37]

**Table 2:** Practical applications of certain antigens in combination with nanomaterials.

Antigen Type	Intrinsic Characteristics	Primary Benefits of Nano-Adjuvant Modification	Representative Nano-Adjuvants	Representative Applications	References
Protein Antigens	Large molecular weight, naturally immunogenic but prone to degradation	Stabilizes antigen structure, enhances uptake, prolongs immune persistence	Nano-aluminum salts (e.g., nano-Al(OH)_3_), ISCOMs	Influenza vaccines, Hepatitis B vaccines, DTaP vac-cines	[51,52,53]
Peptide Antigens	Small molecules with weak immunogenicity	Promotes cellular uptake, enhances cross-presentation, activates CTL responses	ISCOMs, PLGA nanoparticles, cationic nanoemulsions	Tuberculosis vac-cines, malaria vaccines	[54,55,56]
Nucleic Acid Antigens	Easily degraded, requires intracellular expression	Improves stability, facilitates intracellular delivery and translational expression	LNPs, polymeric carriers (e.g., PEI, chitosan), nanoemulsions	COVID-19 mRNA vaccines, DNA vaccines	[57,58]

**Table 3:** Vaccines using conventional adjuvants.

Name	Adjuvant Used	Trial Phase	Reference
SCB-2019	CpG/Alum adjuvants	Phase III Clinical Trial	[165]
MAS-1	A novel emulsion adjuvant	Phase I Clinical Trial	[166]
ChAdOx1 nCoV-19	Matrix-M	Phase II Clinical Trial	[167]
Cecolin	ALOOH	Phase III Clinical Trial	[168]
CoV2 preS dTM-AS03 Monovalent (D614)	AS03	Phase III Clinical Trial	[169]
CIMAVAX-EGF	Montanide ISA 51	Phase III Clinical Trial	[170]

## Data Availability

No new data were created or analyzed in this study. Data sharing is not applicable to this article.

## References

[1] Trautmann C., Brüchle W., Spohr R., Vetter J., Angert N. (1996). Pore geometry of etched ion tracks in polyimide. Nucl. Instrum. Methods Phys. Res. Sect. B.

[2] Gerhard W., Mozdzanowska K., Zharikova D. (2006). Prospects for Universal Influenza Virus Vaccine. Emerg. Infect. Dis..

[3] Sterner E. (2024). Analyses of the 2023 Nobel Prize in Physiology or Medicine: Nucleoside Base Modifications and Effective mRNA Vaccines. Sci. Technol. Libr..

[4] Ghattas M., Dwivedi G., Lavertu M., Alameh M.-G. (2021). Vaccine Technologies and Platforms for Infectious Diseases: Current Progress, Challenges, and Opportunities. Vaccines.

[5] Xue J.-B., Lai D.-Y., Jiang H.-W., Qi H., Guo S.-J., Zhu Y.-S., Xu H., Zhou J., Tao S.-C. (2022). Landscape of the RBD-specific IgG, IgM, and IgA responses triggered by the inactivated virus vaccine against the Omicron variant. Cell Discov..

[6] Chen X., Chen Z., Azman A.S., Sun R., Lu W., Zheng N., Zhou J., Wu Q., Deng X., Zhao Z. (2021). Neutralizing Antibodies Against Severe Acute Respiratory Syndrome Coronavirus 2 (SARS-CoV-2) Variants Induced by Natural Infection or Vaccination: A Systematic Review and Pooled Analysis. Clin. Infect. Dis..

[7] Klein N.P., Fireman B., Yih W.K., Lewis E., Kulldorff M., Ray P., Baxter R., Hambidge S., Nordin J., Naleway A. (2010). Measles-Mumps-Rubella-Varicella Combination Vaccine and the Risk of Febrile Seizures. Pediatrics.

[8] Schink T., Holstiege J., Kowalzik F., Zepp F., Garbe E. (2014). Risk of febrile convulsions after MMRV vaccination in comparison to MMR or MMR+V vaccination. Vaccine.

[9] Semenzato L., Le Vu S., Botton J., Bertrand M., Jabagi M.-J., Drouin J., Cuenot F., Zores F., Dray-Spira R., Weill A. (2024). Long-Term Prognosis of Patients With Myocarditis Attributed to COVID-19 mRNA Vaccination, SARS-CoV-2 Infection, or Conventional Etiologies. JAMA.

[10] Pires-Marczeski F.C., Martinez V.P., Nemirovsky C., Padula P.J. (2011). Intrathecal antibody production in two cases of yellow fever vaccine associated neurotropic disease in Argentina. J. Med. Virol..

[11] Schillie S., Vellozzi C., Reingold A., Harris A., Haber P., Ward J.W., Nelson N.P. (2018). Prevention of Hepatitis B Virus Infection in the United States: Recommendations of the Advisory Committee on Immunization Practices. MMWR Recomm. Rep..

[12] Xiao C., Ren Z., Zhang B., Mao L., Zhu G., Gao L., Su J., Ye J., Long Z., Zhu Y. (2023). Insufficient epitope-specific T cell clones are responsible for impaired cellular immunity to inactivated SARS-CoV-2 vaccine in older adults. Nat. Aging.

[13] Park J.-E., Jardine L., Gottgens B., Teichmann S.A., Haniffa M. (2020). Prenatal development of human immunity. Science.

[14] Scheiblhofer S., Laimer J., Machado Y., Weiss R., Thalhamer J. (2017). Influence of protein fold stability on immunogenicity and its implications for vaccine design. Exp. Rev. Vaccines.

[15] Foged C. (2011). Subunit Vaccines of the Future: The Need for safe, Customized and Optimized Particulate Delivery Systems. Ther. Deliv..

[16] Heidegger S., Gößl D., Schmidt A., Niedermayer S., Argyo C., Endres S., Bein T., Bourquin C. (2016). Immune response to functionalized mesoporous silica nanoparticles for targeted drug delivery. Nanoscale.

[17] Kim C.-G., Lee J.-C., Ju D.-B., Kim S.-K., Yun C.-H., Cho C.-S. (2023). Enhancement of Immune Responses Elicited by Nanovaccines through a Cross-Presentation Pathway. Tissue Eng. Regener. Med..

[18] Farasati Far B., Naimi-Jamal M.R., Safaei M., Zarei K., Moradi M., Yazdani Nezhad H. (2022). A Review on Biomedical Application of Polysaccharide-Based Hydrogels with a Focus on Drug Delivery Systems. Polymers.

[19] Hou Y., Chen M., Bian Y., Hu Y., Chuan J., Zhong L., Zhu Y., Tong R. (2024). Insights into vaccines for elderly individuals: From the impacts of immunosenescence to delivery strategies. NPJ Vaccines.

[20] Cao X. (2016). Self-regulation and cross-regulation of pattern-recognition receptor signalling in health and disease. Nat. Rev. Immunol..

[21] Carroll S.L., Pasare C., Barton G.M. (2024). Control of adaptive immunity by pattern recognition receptors. Immunity.

[22] Martiñón S., Cisneros A., Villicaña S., Hernández-Miramontes R., Mixcoha E., Calderón-Vargas P. (2019). Chemical and Immunological Characteristics of Aluminum-Based, Oil-Water Emulsion, and Bacterial-Origin Adjuvants. J. Immunol. Res..

[23] Yuan L., Gao X.-D., Xia Y. (2022). Optimising the oil phases of aluminium hydrogel-stabilised emulsions for stable, safe and efficient vaccine adjuvant. Front. Chem. Sci. Eng..

[24] Martina B.E.E., van de Bildt M.W.G., Kuiken T., van Amerongen G., Osterhaus A.D.M.E. (2003). Immunogenicity and efficacy of recombinant subunit vaccines against phocid herpesvirus type 1. Vaccine.

[25] Ulmer J. (2013). Vaccine Adjuvants: Mode of Action. Front. Immunol..

[26] Pedersen G.K., Wørzner K., Andersen P., Christensen D. (2020). Vaccine Adjuvants Differentially Affect Kinetics of Antibody and Germinal Center Responses. Front. Immunol..

[27] Fox C.B., Kramer R.M., Barnes V.L., Dowling Q.M., Vedvick T.S. (2013). Working together: Interactions between vaccine antigens and adjuvants. Ther. Adv. Vaccines.

[28] Cui Y., Ho M., Hu Y., Shi Y. (2024). Vaccine adjuvants: Current status, research and development, licensing, and future opportunities. J. Mater. Chem. B.

[29] Qiao D., Li L., Liu L., Chen Y. (2022). Universal and Translational Nanoparticulate CpG Adjuvant. ACS Appl. Mater. Interfaces.

[30] Xu L., Liu Y., Chen Z., Li W., Liu Y., Wang L., Ma L., Shao Y., Zhao Y., Chen C. (2013). Morphologically Virus-Like Fullerenol Nanoparticles Act as the Dual-Functional Nanoadjuvant for HIV-1 Vaccine. Adv. Mater..

[31] Moni S.S., Abdelwahab S.I., Jabeen A., Elmobark M.E., Aqaili D., Gohal G., Oraibi B., Farasani A.M., Jerah A.A., Alnajai M.M.A. (2023). Advancements in Vaccine Adjuvants: The Journey from Alum to Nano Formulations. Vaccines.

[32] Barbateskovic M., Klingenberg S.L., Krauss S.R., Kong D., Wu Z., Petersen S.B., Kenfelt M., Gluud C. (2023). Concentrations, Number of Doses, and Formulations of Aluminium Adjuvants in Vaccines: A Systematic Review with Meta-Analysis and Trial Sequential Analysis of Randomized Clinical Trials. Vaccines.

[33] Laera D., HogenEsch H., O’Hagan D.T. (2023). Aluminum Adjuvants—‘Back to the Future’. Pharmaceutics.

[34] O’Hagan D.T., Ott G.S., Van Nest G. (1997). Recent advances in vaccine adjuvants: The development of MF59 emulsion and polymeric microparticles. Mol. Med. Today.

[35] Cohet C., van der Most R., Bauchau V., Bekkat-Berkani R., Doherty T.M., Schuind A., Tavares Da Silva F., Rappuoli R., Garçon N., Innis B.L. (2019). Safety of AS03-adjuvanted influenza vaccines: A review of the evidence. Vaccine.

[36] Didierlaurent A.M., Laupèze B., Di Pasquale A., Hergli N., Collignon C., Garçon N. (2017). Adjuvant system AS01: Helping to overcome the challenges of modern vaccines. Exp. Rev. Vaccines.

[37] Hoxie I., Vasilev K., Clark J.J., Bushfield K., Francis B., Loganathan M., Campbell J.D., Yu D., Guan L., Gu C. (2024). A recombinant N2 neuraminidase-based CpG 1018^®^ adjuvanted vaccine provides protection against challenge with heterologous influenza viruses in mice and hamsters. Vaccine.

[38] Li Y., Wu W., Liu Q., Wu Q., Ren P., Xi X., Liu H., Zhao J., Zhang W., Wang Z. (2024). Specific surface-modified iron oxide nanoparticles trigger complement-dependent innate and adaptive antileukaemia immunity. Nat. Commun..

[39] Croitoru G.-A., Pîrvulescu D.-C., Niculescu A.-G., Epistatu D., Rădulescu M., Grumezescu A.M., Nicolae C.-L. (2024). Nanomaterials in Immunology: Bridging Innovative Approaches in Immune Modulation, Diagnostics, and Therapy. J. Funct. Biomater..

[40] Dagan N., Barda N., Kepten E., Miron O., Perchik S., Katz M.A., Hernán M.A., Lipsitch M., Reis B., Balicer R.D. (2021). BNT162b2 mRNA Covid-19 Vaccine in a Nationwide Mass Vaccination Setting. N. Engl. J. Med..

[41] Liu J., Liu Y., Xia H., Zou J., Weaver S.C., Swanson K.A., Cai H., Cutler M., Cooper D., Muik A. (2021). BNT162b2-elicited neutralization of B.1.617 and other SARS-CoV-2 variants. Nature.

[42] Gu Y., Chen J., Wang Z., Liu C., Wang T., Kim C.-J., Durikova H., Fernandes S., Johnson D.N., De Rose R. (2024). mRNA delivery enabled by metal–organic nanoparticles. Nat. Commun..

[43] Zeng Y., Estapé Senti M., Labonia M.C.I., Papadopoulou P., Brans M.A.D., Dokter I., Fens M.H., van Mil A., Sluijter J.P.G., Schiffelers R.M. (2023). Fusogenic Coiled-Coil Peptides Enhance Lipid Nanoparticle-Mediated mRNA Delivery upon Intramyocardial Administration. ACS Nano.

[44] Panagiotakopoulos L., Moulia D.L., Godfrey M., Link-Gelles R., Roper L., Havers F.P., Taylor C.A., Stokley S., Talbot H.K., Schechter R. (2024). Use of COVID-19 Vaccines for Persons Aged ≥6 Months: Recommendations of the Advisory Committee on Immunization Practices—United States, 2024–2025. MMWR Morb. Mortal. Wkly. Rep..

[45] Olshefsky A., Richardson C., Pun S.H., King N.P. (2022). Engineering Self-Assembling Protein Nanoparticles for Therapeutic Delivery. Bioconjugate Chem..

[46] Ikwuagwu B., Tullman-Ercek D. (2022). Virus-like particles for drug delivery: A review of methods and applications. Curr. Opin. Biotechnol..

[47] Yang J.-X., Tseng J.-C., Yu G.-Y., Luo Y., Huang C.-Y.F., Hong Y.-R., Chuang T.-H. (2022). Recent Advances in the Development of Toll-like Receptor Agonist-Based Vaccine Adjuvants for Infectious Diseases. Pharmaceutics.

[48] Facciolà A., Visalli G., Laganà A., Di Pietro A. (2022). An Overview of Vaccine Adjuvants: Current Evidence and Future Perspectives. Vaccines.

[49] Zhao T., Cai Y., Jiang Y., He X., Wei Y., Yu Y., Tian X. (2023). Vaccine adjuvants: Mechanisms and platforms. Signal Transduct. Target. Ther..

[50] Mochida Y., Uchida S. (2024). mRNA vaccine designs for optimal adjuvanticity and delivery. RNA Biol..

[51] Wang S.H., Smith D., Cao Z., Chen J., Acosta H., Chichester J.A., Yusibov V., Streatfield S.J., Fattom A., Baker J.R. (2019). Recombinant H5 hemagglutinin adjuvanted with nanoemulsion protects ferrets against pathogenic avian influenza virus challenge. Vaccine.

[52] Zhang T., He P., Guo D., Chen K., Hu Z., Zou Y. (2023). Research Progress of Aluminum Phosphate Adjuvants and Their Action Mechanisms. Pharmaceutics.

[53] Gogoi H., Rajesh M., Bhatnagar R. (2025). Re-inventing traditional aluminum-based adjuvants: Insight into a century of advancements. Int. Rev. Immunol..

[54] Hassan H.A.F.M., Haider M., Fahmy S.A. (2024). From antigen uptake to immune modulation: The multifaceted potential of peptide nanofibers as vaccine nanocarriers. Mater. Adv..

[55] Sun B., Ji Z., Liao Y.-P., Chang C.H., Wang X., Ku J., Xue C., Mirshafiee V., Xia T. (2017). Enhanced Immune Adjuvant Activity of Aluminum Oxyhydroxide Nanorods through Cationic Surface Functionalization. ACS Appl. Mater. Interfaces.

[56] Büyükbayraktar H.K., Pelit Arayıcı P., Ihlamur M., Gökkaya D., Karahan M., Abamor E.Ş., Topuzoğulları M. (2023). Effect of polycation coating on the long-term pulsatile release of antigenic ESAT-61–20 peptide from PLGA nanoparticles. Colloids Surf. B.

[57] Liang Z., Bao H., Yao Z., Li M., Chen C., Zhang L., Wang H., Guo Y., Ma Y., Yang X. (2024). The orientation of CpG conjugation on aluminum oxyhydroxide nanoparticles determines the immunostimulatory effects of combination adjuvants. Biomaterials.

[58] Hou X., Zaks T., Langer R., Dong Y. (2021). Lipid nanoparticles for mRNA delivery. Nat. Rev. Mater..

[59] Lee D., Huntoon K., Lux J., Kim B.Y.S., Jiang W. (2023). Engineering nanomaterial physical characteristics for cancer immunotherapy. Nat. Rev. Bioeng..

[60] Fernández-Tejada A., Chea E.K., George C., Pillarsetty N., Gardner J.R., Livingston P.O., Ragupathi G., Lewis J.S., Tan D.S., Gin D.Y. (2014). Development of a minimal saponin vaccine adjuvant based on QS-21. Nat. Chem..

[61] Cruz-Bustos T., González-González G., Morales-Sanfrutos J., Megía-Fernández A., Santoyo-González F., Osuna A. (2012). Functionalization of immunostimulating complexes (ISCOMs) with lipid vinyl sulfones and their application in immunological techniques and therapy. Int. J. Nanomed..

[62] Glenny A.T. (1926). The antigenic value of toxoid precipitated by potassium alum. J. Pathol. Bacteriol..

[63] HogenEsch H. (2013). Mechanism of Immunopotentiation and Safety of Aluminum Adjuvants. Front. Immunol..

[64] Pulendran B.S., Arunachalam P., O’Hagan D.T. (2021). Emerging concepts in the science of vaccine adjuvants. Nat. Rev. Drug Discov..

[65] Hem S.L., Johnston C.T. (2014). Production and Characterization of Aluminum-Containing Adjuvants. Vaccine Development and Manufacturing.

[66] Shardlow E., Mold M., Exley C. (2018). Unraveling the enigma: Elucidating the relationship between the physicochemical properties of aluminium-based adjuvants and their immunological mechanisms of action. Allergy Asthma Clin. Immunol..

[67] Rinella J.V., White J.L., Hem S.L. (1995). Effect of Anions on Model Aluminum-Adjuvant-Containing Vaccines. J. Colloid. Interface Sci..

[68] Lu Y., Liu G. (2022). Nano alum: A new solution to the new challenge. Hum. Vaccines Immunother..

[69] Liang Z., Wang X., Yu G., Li M., Shi S., Bao H., Chen C., Fu D., Ma W., Xue C. (2022). Mechanistic understanding of the aspect ratio-dependent adjuvanticity of engineered aluminum oxyhydroxide nanorods in prophylactic vaccines. Nano Today.

[70] Chen W., Zuo H., Li B., Duan C., Rolfe B., Zhang B., Mahony T.J., Xu Z.P. (2018). Clay Nanoparticles Elicit Long-Term Immune Responses by Forming Biodegradable Depots for Sustained Antigen Stimulation. Small.

[71] Chen W., Zhang B., Mahony T., Gu W., Rolfe B., Xu Z.P. (2016). Efficient and Durable Vaccine against Intimin β of Diarrheagenic *E. coli* Induced by Clay Nanoparticles. Small.

[72] Ruwona T.B., Xu H., Li X., Taylor A.N., Shi Y.-c., Cui Z. (2016). Toward understanding the mechanism underlying the strong adjuvant activity of aluminum salt nanoparticles. Vaccine.

[73] Orr M.T., Khandhar A.P., Seydoux E., Liang H., Gage E., Mikasa T., Beebe E.L., Rintala N.D., Persson K.H., Ahniyaz A. (2019). Reprogramming the adjuvant properties of aluminum oxyhydroxide with nanoparticle technology. NPJ Vaccines.

[74] Thakkar S.G., Xu H., Li X., Cui Z. (2018). Uric acid and the vaccine adjuvant activity of aluminium (oxy)hydroxide nanoparticles. J. Drug Target..

[75] Vrieling H., Espitia Ballestas M., Hamzink M., Willems G.-J., Soema P., Jiskoot W., Kersten G., Metz B. (2019). Stabilised aluminium phosphate nanoparticles used as vaccine adjuvant. Colloids Surf. B.

[76] Lebre F., Pedroso de Lima M.C., Lavelle E.C., Borges O. (2018). Mechanistic study of the adjuvant effect of chitosan-aluminum nanoparticles. Int. J. Pharm..

[77] Wang X., Cao F., Yan M., Liu Y., Zhu X., Sun H., Ma G. (2019). Alum-functionalized graphene oxide nanocomplexes for effective anticancer vaccination. Acta Biomater..

[78] Jiang H., Wang Q., Li L., Zeng Q., Li H., Gong T., Zhang Z., Sun X. (2018). Turning the Old Adjuvant from Gel to Nanoparticles to Amplify CD8+ T Cell Responses. Adv. Sci..

[79] Li D., Xu M., Li G., Zheng Y., Zhang Y., Xia D., Wang S., Chen Y. (2022). Mg/Al-LDH as a nano-adjuvant for pertussis vaccine: A evaluation compared with aluminum hydroxide adjuvant. Nanotechnology.

[80] Sun B., Ji Z., Liao Y.-P., Wang M., Wang X., Dong J., Chang C.H., Li R., Zhang H., Nel A.E. (2013). Engineering an Effective Immune Adjuvant by Designed Control of Shape and Crystallinity of Aluminum Oxyhydroxide Nanoparticles. ACS Nano.

[81] Zhu M., Wang R., Nie G. (2014). Applications of nanomaterials as vaccine adjuvants. Hum. Vaccines Immunother..

[82] Mehdi K., Mohsen M., Naser Mohammadpour D., Mohsen M., Alireza M. (2020). Nanoparticles and Vaccine Development. Pharm. Nanotechnol..

[83] Sun B., Xia T. (2016). Nanomaterial-based vaccine adjuvants. J. Mater. Chem. B.

[84] Nazarizadeh A., Staudacher A.H., Wittwer N.L., Turnbull T., Brown M.P., Kempson I. (2022). Aluminium Nanoparticles as Efficient Adjuvants Compared to Their Microparticle Counterparts: Current Progress and Perspectives. Int. J. Mol. Sci..

[85] Ghahary M., Taheri R.A., Fasihi-Ramandi M.J.J.o.M.U.o.M.S. (2019). Protective Efficacy of the Killed Toxoplasma gondii Vaccine in Nano-alum Adjuvant. J. Maz. Univ. Med. Sci..

[86] Bi S., Li M., Liang Z., Li G., Yu G., Zhang J., Chen C., Yang C., Xue C., Zuo Y.Y. (2022). Self-assembled aluminum oxyhydroxide nanorices with superior suspension stability for vaccine adjuvant. J. Colloid. Interface Sci..

[87] Mallakpour S., Nikkhoo E., Hussain C.M. (2022). Application of MOF materials as drug delivery systems for cancer therapy and dermal treatment. Coord. Chem. Rev..

[88] Ma D., Wang G., Lu J., Zeng X., Cheng Y., Zhang Z., Lin N., Chen Q. (2023). Multifunctional nano MOF drug delivery platform in combination therapy. Eur. J. Med. Chem..

[89] Zhong X., Zhang Y., Tan L., Zheng T., Hou Y., Hong X., Du G., Chen X., Zhang Y., Sun X. (2019). An aluminum adjuvant-integrated nano-MOF as antigen delivery system to induce strong humoral and cellular immune responses. J. Control. Release.

[90] Liu J., Guo S., Jin Z., Zhao K. (2023). Adjuvanted quaternized chitosan composite aluminum nanoparticles-based vaccine formulation promotes immune responses in chickens. Vaccine.

[91] Sokal E.M., Hoppenbrouwers K., Vandermeulen C., Moutschen M., Léonard P., Moreels A., Haumont M., Bollen A., Smets F., Denis M. (2007). Recombinant gp350 Vaccine for Infectious Mononucleosis: A Phase 2, Randomized, Double- Blind, Placebo-Controlled Trial to Evaluate the Safety, Immunogenicity, and Efficacy of an Epstein- Barr Virus Vaccine in Healthy Young Adults. J. Infect. Dis..

[92] Garçon N., Morel S., Didierlaurent A., Descamps D., Wettendorff M., Van Mechelen M. (2011). Development of an AS04-Adjuvanted HPV Vaccine with the Adjuvant System Approach. BioDrugs.

[93] Knop K., Hoogenboom R., Fischer D., Schubert U.S. (2010). Poly(ethylene glycol) in Drug Delivery: Pros and Cons as Well as Potential Alternatives. Angew. Chem. Int. Ed..

[94] He C., He P., Tang X., Bai S., Qin M., Zhang Y., Guo Z., Du G., Sun X. (2025). Zoledronate-loaded aluminum salt nanovaccines amplify cellular immune response by enhancing cross-presentation. Nano Res..

[95] Callahan P.M., Shorter A.L., Hem S.L. (1991). The Importance of Surface Charge in the Optimization of Antigen–Adjuvant Interactions. Pharm. Res..

[96] Yu G., Liang Z., Yu Z., Li M., Yang W., Zhang Y., Zhao Y., Yang C., Xue C., Shi L. (2022). Engineering the hydroxyl content on aluminum oxyhydroxide nanorod for elucidating the antigen adsorption behavior. NPJ Vaccines.

[97] Chen D., Ling X., Wang Y., Zhang Q., He X., Dong Z., Li M., He Q. (2025). Autophagy-activating aluminum hydroxide nanovaccine for enhanced antigen presentation and antitumor immunity. J. Control. Release.

[98] Hood M.I., Skaar E.P. (2012). Nutritional immunity: Transition metals at the pathogen–host interface. Nat. Rev. Microbiol..

[99] Wang C., Zhang R., Wei X., Lv M., Jiang Z., Dong C., Jiang Z. (2020). Chapter Seven—Metalloimmunology: The metal ion-controlled immunity. Advances in Immunology.

[100] Li Y., Wang C., Lv H., Li J., Zhang X., Zhang S., Shen Q., Wu Q., Liu Y., Peng R. (2024). Manganese-Modified Aluminum Adjuvant Enhances both Humoral and Cellular Immune Responses. Adv. Healthc. Mater..

[101] Liu D., Liang S., Ma K., Meng Q.-F., Li X., Wei J., Zhou M., Yun K., Pan Y., Rao L. (2024). Tumor Microenvironment-Responsive Nanoparticles Amplifying STING Signaling Pathway for Cancer Immunotherapy. Adv. Mater..

[102] Sjölander A., Cox J.C. (1998). Uptake and adjuvant activity of orally delivered saponin and ISCOM™ vaccines. Adv. Drug Deliv. Rev..

[103] Barr I.G., Sjölander A., Cox J.C. (1998). ISCOMs and other saponin based adjuvants. Adv. Drug Deliv. Rev..

[104] Zhu D., Tuo W. (2016). QS-21: A Potent Vaccine Adjuvant. Nat. Prod. Chem. Res..

[105] Marty-Roix R., Vladimer G.I., Pouliot K., Weng D., Buglione-Corbett R., West K., MacMicking J.D., Chee J.D., Wang S., Lu S. (2016). Identification of QS-21 as an Inflammasome-activating Molecular Component of Saponin Adjuvants. J. Biol. Chem..

[106] Pal S., Nath S., Meininger C.J., Gashev A.A. (2020). Emerging Roles of Mast Cells in the Regulation of Lymphatic Immuno-Physiology. Front. Immunol..

[107] Silva M., Kato Y., Melo M.B., Phung I., Freeman B.L., Li Z., Roh K., Van Wijnbergen J.W., Watkins H., Enemuo C.A. (2021). A particulate saponin/TLR agonist vaccine adjuvant alters lymph flow and modulates adaptive immunity. Sci. Immunol..

[108] Morein B., Lövgren K., Höglund S., Sundquist B. (1987). The ISCOM: An immunostimulating complex. Immunol. Today.

[109] Morein B., Lövgren K., Rönnberg B., Sjölander A., Villacrés-Eriksson M. (1995). Immunostimulating Complexes. Clin. Immunother..

[110] Nielsen H.M., Hübschmann H.B., Rades T., Foged C., Rades T., Perrie Y., Hook S. (2015). ISCOMs as a Vaccine Delivery System. Subunit Vaccine Delivery.

[111] Cuevas-Romero J.S., Cerriteño-Sánchez J.L., Lara-Romero R., Vega-López M.A., Ramírez-Estudillo C., Ramírez-Mendoza H., Berg M., Lövgren-Bengtsson K. (2022). Immunogenicity of a recombinant hemagglutinin neuraminidase-Porcine rubulavirus produced by Escherichia coli of Porcine rubulavirus gives protective immunity of litter after challenge. J. Vet. Med. Sci..

[112] Chen K., Wang N., Zhang X., Wang M., Liu Y., Shi Y. (2023). Potentials of saponins-based adjuvants for nasal vaccines. Front. Immunol..

[113] Zhu W., Park J., Pho T., Wei L., Dong C., Kim J., Ma Y., Champion J.A., Wang B.-Z. (2023). ISCOMs/MPLA-Adjuvanted SDAD Protein Nanoparticles Induce Improved Mucosal Immune Responses and Cross-Protection in Mice. Small.

[114] Pires I.S., Ni K., Melo M.B., Li N., Ben-Akiva E., Maiorino L., Dye J., Rodrigues K.A., Yun D., Kim B. (2023). Controlled lipid self-assembly for scalable manufacturing of next-generation immune stimulating complexes. Chem. Eng. J..

[115] White K., Rades T., Kearns P., Toth I., Hook S. (2006). Immunogenicity of Liposomes Containing Lipid Core Peptides and the Adjuvant Quil A. Pharm. Res..

[116] Myschik J., McBurney W.T., Hennessy T., Phipps-Green A., Rades T., Hook S. (2008). Immunostimulatory biodegradable implants containing the adjuvant Quil-A—Part II: In vivo evaluation. J. Drug Target..

[117] Heath P.T., Galiza E.P., Baxter D.N., Boffito M., Browne D., Burns F., Chadwick D.R., Clark R., Cosgrove C.A., Galloway J. (2022). Safety and Efficacy of the NVX-CoV2373 Coronavirus Disease 2019 Vaccine at Completion of the Placebo-Controlled Phase of a Randomized Controlled Trial. Clin. Infect. Dis..

[118] Kersten G.F., Teerlink T., Derks H.J., Verkleij A.J., Wezel T.L.v., Crommelin D.J., Beuvery E.C. (1988). Incorporation of the major outer membrane protein of Neisseria gonorrhoeae in saponin-lipid complexes (iscoms): Chemical analysis, some structural features, and comparison of their immunogenicity with three other antigen delivery systems. Infect Immun..

[119] Demana P.H., Fehske C., White K., Rades T., Hook S. (2004). Effect of incorporation of the adjuvant Quil A on structure and immune stimulatory capacity of liposomes. Immunol. Cell Biol..

[120] Lövgren Bengtsson K., Morein B., Osterhaus A.D.M.E. (2011). ISCOM technology-based Matrix M™ adjuvant: Success in future vaccines relies on formulation. Exp. Rev. Vaccines.

[121] Stertman L., Palm A.-K.E., Zarnegar B., Carow B., Lunderius Andersson C., Magnusson S.E., Carnrot C., Shinde V., Smith G., Glenn G. (2023). The Matrix-M™ adjuvant: A critical component of vaccines for the 21st century. Hum. Vaccines Immunother..

[122] Heath P.T., Galiza E.P., Baxter D.N., Boffito M., Browne D., Burns F., Chadwick D.R., Clark R., Cosgrove C., Galloway J. (2021). Safety and Efficacy of NVX-CoV2373 Covid-19 Vaccine. N. Engl. J. Med..

[123] Keech C., Albert G., Cho I., Robertson A., Reed P., Neal S., Plested J.S., Zhu M., Cloney-Clark S., Zhou H. (2020). Phase 1–2 Trial of a SARS-CoV-2 Recombinant Spike Protein Nanoparticle Vaccine. N. Engl. J. Med..

[124] Dunkle L.M., Kotloff K.L., Gay C.L., Áñez G., Adelglass J.M., Hernández A.Q.B., Harper W.L., Duncanson D.M., McArthur M.A., Florescu D.F. (2022). Efficacy and Safety of NVX-CoV2373 in Adults in the United States and Mexico. N. Engl. J. Med..

[125] Hook S., Rades T., Flower D.R., Perrie Y. (2013). Immune Stimulating Complexes (ISCOMs) and Quil-A Containing Particulate Formulations as Vaccine Delivery Systems. Immunomic Discovery of Adjuvants and Candidate Subunit Vaccines.

[126] Li I.A., Popov A.M., Sanina N.M., Kostetskii E.Y., Novikova O.D., Reunov A.V., Nagorskaya V.P., Portnyagina O.Y., Khomenko V.A., Shnyrov V.L. (2004). Physicochemical and Immune Properties of Glycoglycerolipids from Laminaria japonicain Immunostimulating Complexes (ISCOMs). Biol. Bull. Russ. Acad. Sci..

[127] Lendemans D.G., Egert A.M., Hook S., Rades T. (2007). Cage-like complexes formed by DOTAP, Quil-A and cholesterol. Int. J. Pharm..

[128] Lendemans D.G., Myschik J., Hook S., Rades T. (2005). Cationic cage-like complexes formed by DC-cholesterol, Quil-A, and phospholipid. J. Pharm. Sci..

[129] Höglund S., Dalsgaard K., Lövgren K., Sundquist B., Osterhaus A., Morein B., Harris J.R. (1989). ISCOMs and Immunostimulation with Viral Antigens. Virally Infected Cells.

[130] Morein B., Bengtsson K.L. (1999). Immunomodulation by Iscoms, Immune Stimulating Complexes. Methods.

[131] Morein B., Bengtsson K.L. (1998). Functional aspects of iscoms. Immunol. Cell Biol..

[132] Qiao X., Qu L., Guo Y., Hoshino T. (2021). Secondary Structure and Conformational Stability of the Antigen Residues Making Contact with Antibodies. J. Phys. Chem. B.

[133] Qu L., Qiao X., Qi F., Nishida N., Hoshino T. (2021). Analysis of Binding Modes of Antigen–Antibody Complexes by Molecular Mechanics Calculation. J. Chem. Inf. Model..

[134] Le T.T.T., Drane D., Malliaros J., Cox J.C., Rothel L., Pearse M., Woodberry T., Gardner J., Suhrbier A. (2001). Cytotoxic T cell polyepitope vaccines delivered by ISCOMs. Vaccine.

[135] Buglione-Corbett R., Pouliot K., Marty-Roix R., Li W., West K., Wang S., Morelli A.B., Lien E., Lu S. (2014). Reduced MyD88 dependency of ISCOMATRIX™ adjuvant in a DNA prime-protein boost HIV vaccine. Hum. Vaccines Immunother..

[136] De Libero G., Mori L. (2005). Recognition of lipid antigens by T cells. Nat. Rev. Immunol..

[137] Rao M., Alving C.R. (2000). Delivery of lipids and liposomal proteins to the cytoplasm and Golgi of antigen-presenting cells. Adv. Drug Deliv. Rev..

[138] Moody D.B., Zajonc D.M., Wilson I.A. (2005). Anatomy of CD1–lipid antigen complexes. Nat. Rev. Immunol..

[139] McCluskie M.J., Weeratna R.D., Evans D.M., Makinen S., Drane D., Davis H.L. (2013). CpG ODN and ISCOMATRIX adjuvant: A synergistic adjuvant combination inducing strong T-Cell IFN-γ responses. Biomed. Res. Int..

[140] Ko E.-J., Kang S.-M. (2018). Immunology and efficacy of MF59-adjuvanted vaccines. Hum. Vaccines Immunother..

[141] Sánchez-Quesada C., López-Biedma A., Toledo E., Gaforio J.J. (2018). Squalene Stimulates a Key Innate Immune Cell to Foster Wound Healing and Tissue Repair. Evid. Based Complement. Altern. Med..

[142] Ho H.-M., Huang C.-Y., Cheng Y.-J., Shen K.-Y., Tzeng T.-T., Liu S.-J., Chen H.-W., Huang C.-H., Huang M.-H. (2021). Assessment of adjuvantation strategy of lipid squalene nanoparticles for enhancing the immunogenicity of a SARS-CoV-2 spike subunit protein against COVID-19. Int. J. Pharm..

[143] Valenzuela P., Medina A., Rutter W.J., Ammerer G., Hall B.D. (1982). Synthesis and assembly of hepatitis B virus surface antigen particles in yeast. Nature.

[144] Noel Masihi K., Lange W., Brehmer W., Ribi E. (1986). Immunobiological activities of nontoxic lipid A: Enhancement of nonspecific resistance in combination with trehalose dimycolate against viral infection and adjuvant effects. Int. J. Immunopharmacol..

[145] Allison A.C., Byars N.E. (1986). An adjuvant formulation that selectively elicits the formation of antibodies of protective isotypes and of cell-mediated immunity. J. Immunol. Methods.

[146] Shi S., Liang Z., Sun B. (2020). Response to comment on: Vaccine adjuvants: Understanding the structure and mechanism of adjuvanticity. Vaccine.

[147] O’Hagan D.T., Ott G.S., De Gregorio E., Seubert A. (2012). The mechanism of action of MF59—An innately attractive adjuvant formulation. Vaccine.

[148] Seubert A., Monaci E., Pizza M., O’Hagan D.T., Wack A. (2008). The Adjuvants Aluminum Hydroxide and MF59 Induce Monocyte and Granulocyte Chemoattractants and Enhance Monocyte Differentiation toward Dendritic Cells1. J. Immunol..

[149] O’Hagan D.T. (2007). MF59 is a safe and potent vaccine adjuvant that enhances protection against influenza virus infection. Exp. Rev. Vaccines.

[150] Chappell K.J., Mordant F.L., Li Z., Wijesundara D.K., Ellenberg P., Lackenby J.A., Cheung S.T.M., Modhiran N., Avumegah M.S., Henderson C.L. (2021). Safety and immunogenicity of an MF59-adjuvanted spike glycoprotein-clamp vaccine for SARS-CoV-2: A randomised, double-blind, placebo-controlled, phase 1 trial. Lancet Infect. Dis..

[151] Amarowicz R. (2009). Squalene: A natural antioxidant?. Eur. J. Lipid Sci. Technol..

[152] Lin X., Sheng Y., Zhang X., Li Z., Yang Y., Wu J., Su Z., Ma G., Zhang S. (2022). Oil-in-ionic liquid nanoemulsion-based intranasal delivery system for influenza split-virus vaccine. J. Control. Release.

[153] Wang Z., Shan P., Li S., Wei D., Zhang Z., Hao S., Li W., Wang X., Xu J. (2022). Artificial Nanolipid Droplets with Monolayer Lecithin Membranes and Vitamin E Cores as Vaccine Adjuvants. ACS Appl. Nano Mater..

[154] Fisher K.J., Kinsey R., Mohamath R., Phan T., Liang H., Orr M.T., Lykins W.R., Guderian J.A., Bakken J., Argilla D. (2023). Semi-synthetic terpenoids with differential adjuvant properties as sustainable replacements for shark squalene in vaccine emulsions. NPJ Vaccines.

[155] Chen Z., Hao X., Wang H., Zhong X., Chen X., Zhao Y., Zhang Y., Du G., Sun X. (2022). Smart combination of aluminum hydroxide and MF59 to induce strong cellular immune responses. J. Control. Release.

[156] Xia Y., Wu J., Wei W., Du Y., Wan T., Ma X., An W., Guo A., Miao C., Yue H. (2018). Exploiting the pliability and lateral mobility of Pickering emulsion for enhanced vaccination. Nat. Mater..

[157] Shariatinia Z. (2021). Big family of nano- and microscale drug delivery systems ranging from inorganic materials to polymeric and stimuli-responsive carriers as well as drug-conjugates. J. Drug Deliv. Sci. Technol..

[158] Blanco E., Shen H., Ferrari M. (2015). Principles of nanoparticle design for overcoming biological barriers to drug delivery. Nat. Biotechnol..

[159] Xu J., Gattacceca F., Amiji M. (2013). Biodistribution and Pharmacokinetics of EGFR-Targeted Thiolated Gelatin Nanoparticles Following Systemic Administration in Pancreatic Tumor-Bearing Mice. Mol. Pharm..

[160] Manolova V., Flace A., Bauer M., Schwarz K., Saudan P., Bachmann M.F. (2008). Nanoparticles target distinct dendritic cell populations according to their size. Eur. J. Immunol..

[161] Ott G., Barchfeld G.L., Nest G.V. (1995). Enhancement of humoral response against human influenza vaccine with the simple submicron oil/water emulsion adjuvant MF59. Vaccine.

[162] Dupuis M., McDonald D.M., Ott G. (1999). Distribution of adjuvant MF59 and antigen gD2 after intramuscular injection in mice. Vaccine.

[163] Iyer S., HogenEsch H., Hem S.L. (2003). Relationship between the degree of antigen adsorption to aluminum hydroxide adjuvant in interstitial fluid and antibody production. Vaccine.

[164] Gao X., Gong J., Cai Y., Wang J., Wen J., Peng L., Ji H., Jiang S., Guo D. (2021). Chitosan modified squalene nanostructured lipid carriers as a promising adjuvant for freeze-dried ovalbumin vaccine. Int. J. Biol. Macromol..

[165] Bravo L., Smolenov I., Han H.H., Li P., Hosain R., Rockhold F., Clemens S.A.C., Roa C., Borja-Tabora C., Quinsaat A. (2022). Efficacy of the adjuvanted subunit protein COVID-19 vaccine, SCB-2019: A phase 2 and 3 multicentre, double-blind, randomised, placebo-controlled trial. Lancet.

[166] Gorse G.J., Grimes S., Buck H., Mulla H., White P., Hill H., May J., Frey S.E., Blackburn P. (2022). A phase 1 dose-sparing, randomized clinical trial of seasonal trivalent inactivated influenza vaccine combined with MAS-1, a novel water-in-oil adjuvant/delivery system. Vaccine.

[167] Ewer K.J., Barrett J.R., Belij-Rammerstorfer S., Sharpe H., Makinson R., Morter R., Flaxman A., Wright D., Bellamy D., Bittaye M. (2021). T cell and antibody responses induced by a single dose of ChAdOx1 nCoV-19 (AZD1222) vaccine in a phase 1/2 clinical trial. Nat. Med..

[168] Zaman K., Schuind A.E., Adjei S., Antony K., Aponte J.J., Buabeng P.B.Y., Qadri F., Kemp T.J., Hossain L., Pinto L.A. (2024). Safety and immunogenicity of Innovax bivalent human papillomavirus vaccine in girls 9–14 years of age: Interim analysis from a phase 3 clinical trial. Vaccine.

[169] Dayan G.H., Rouphael N., Walsh S.R., Chen A., Grunenberg N., Allen M., Antony J., Bhate A.S., Beresnev T., Bonaparte M.I. (2023). Efficacy of a monovalent (D614) SARS-CoV-2 recombinant protein vaccine with AS03 adjuvant in adults: A phase 3, multi-country study. eClinicalMedicine.

[170] Rodriguez P.C., Popa X., Martínez O., Mendoza S., Santiesteban E., Crespo T., Amador R.M., Fleytas R., Acosta S.C., Otero Y. (2016). A Phase III Clinical Trial of the Epidermal Growth Factor Vaccine CIMAvax-EGF as Switch Maintenance Therapy in Advanced Non–Small Cell Lung Cancer Patients. Clin. Cancer Res..

[171] Zhao Y., Hou J., Guo L., Zhu S., Hou X., Cao S., Zhou M., Shi J., Li J., Liu K. (2024). DNA-Engineered Degradable Invisibility Cloaking for Tumor-Targeting Nanoparticles. J. Am. Chem. Soc..

[172] Mohammapdour R., Ghandehari H. (2022). Mechanisms of immune response to inorganic nanoparticles and their degradation products. Adv. Drug Deliv. Rev..

[173] Heneweer C., Gendy S.E.M., Peñate-Medina O. (2012). Liposomes and Inorganic Nanoparticles for Drug Delivery and Cancer Imaging. Ther. Deliv..

[174] Kisby T., Yilmazer A., Kostarelos K. (2021). Reasons for success and lessons learnt from nanoscale vaccines against COVID-19. Nat. Nanotechnol..

[175] Aljabali A.A., Obeid M.A., Bashatwah R.M., Serrano-Aroca Á., Mishra V., Mishra Y., El-Tanani M., Hromić-Jahjefendić A., Kapoor D.N., Goyal R. (2023). Nanomaterials and Their Impact on the Immune System. Int. J. Mol. Sci..

[176] Ahmed M., Kurungottu P., Swetha K., Atla S., Ashok N., Nagamalleswari E., Bonam S.R., Sahu B.D., Kurapati R. (2025). Role of NLRP3 inflammasome in nanoparticle adjuvant-mediated immune response. Biomater. Sci..

